# 915. Self-esteem Impacted by Scars and Fear of Future Reconstructive Surgeries in Female Burn-injured Adolescents

**DOI:** 10.1093/jbcr/irag033.440

**Published:** 2026-04-06

**Authors:** Ruth B Rimmer, Curt C Bay, Daniel W Chacon, Erika D Mendoza, Nicole Cuevas, Kevin N Foster

**Affiliations:** Care Plans For Life, Phoenix, AZ; AT Still University, Phoenix, AZ; Phoenix Society for Burn Survivors, Fresno, CA; Alisa Ann Ruch Burn Foundation, Fresno, CA; Arizona Burn Foundation, Phoenix, AZ; Diane & Bruce Halle Arizona Burn Center - Valleywise Health, Phoenix, AZ

## Abstract

**Introduction:**

Self-esteem in burn-injured adolescents is a complex yet important issue that intersects with multiple aspects of their recovery and development. These areas include their physical healing, appearance, psychological resilience, social and family support, and the formation of their personal identity. The objective of this study was to determine if self-reported self-esteem among burn-injured youth is correlated with commonly reported burn-related concerns.

**Methods:**

Burn-injured youth were invited to voluntarily complete the 10-item Rosenberg Self Esteem Scale (RSE), which uses responses ranging from "Strongly Agree" to "Strongly Disagree." In addition, they completed a 7-item survey titled "Things Burn-Injured Youth Report Bothering Them about Their Burns," which was developed based on issues noted in the literature. The RSE is widely used for measuring self-esteem, and was originally designed for high school students. A higher score indicates greater self-esteem with 30 as the top score.

**Results:**

A total of 64 burn-injured youth participated in the study. The mean age 15, with a standard deviation of ±3.96 years. The group consisted of 45% females and 55% males, with 17% identifying as Caucasian (n = 11) and 83% as Minority/Other (n = 53) and 75% reporting visible scars. The combined gender RSE score was 20.66 (±5.60), which reflects moderate self-esteem when compared to normative data. Males had an RSE score of 21.26 (±5.71), indicating moderately high self-esteem ranging from below average to high. Females had a mean score of 19.93 (±5.48), just below the midpoint, suggesting moderate average self-esteem, also with a wide range. Importantly, two items were found to be negatively correlated with Rosenberg scores among females: those who were more bothered by the prospect of further surgery reported lower self-esteem (R = -0.417, *p*=.024), and the more their scars bothered them, the lower their self-esteem scores (R = -0.497, *p*=.006).

**Conclusions:**

It is encouraging that, overall, the group reported moderate self-esteem, suggesting that the impact of a burn injury on survivors’ self-esteem may be somewhat limited. However, consistent with findings from other studies, female adolescents reporting challenges associated with their burns reported experiencing lower self-esteem. This population should be assessed by burn care providers to determine whether interventions aimed at improving self-esteem are warranted.

**Applicability of Research to Practice:**

Burn care professionals should consider dedicating time to explaining the benefits of reconstructive surgeries to their adolescent female patients. They should emphasize how restorative surgeries can improve appearance and function, with the goal of enhancing self-esteem.

**Funding for the study:**

Arizona Burn Foundation.

